# Infrared Study of Er^3+^/Yb^3+^ Co-Doped GeO_2_-PbO-Bi_2_O_3_ Glass

**DOI:** 10.3390/ijms13078609

**Published:** 2012-07-10

**Authors:** Hamid-Reza Bahari, Hj. A. A. Sidek, Faisal Rafiq M. Adikan, Wan M. M. Yunus, Mohamed K. Halimah

**Affiliations:** 1 Department of Physics, Faculty of Science, Universiti Putra Malaysia, Serdang, Selangor, UPM 43400, Malaysia; E-Mails: mahmood@science.upm.edu.my (W.M.M.Y.); halimah@science.upm.edu.my (M.K.H.); 2 Department of Electrical Engineering, Faculty of Engineering, University of Malaya, Kuala Lumpur, 50603, Malaysia; E-Mail: rafiq@um.edu.my

**Keywords:** germanate glass, Bi_2_O_3_, PbO, FTIR spectroscopy

## Abstract

Heavy metal oxide glasses, containing bismuth and/or lead in their glass structure are new alternatives for rare eart (RE) doped hosts. Hence, the study of the structure of these vitreous systems is of great interest for science and technology. In this research work, GeO_2_-PbO-Bi_2_O_3_ glass host doped with Er^3+^/Yb^3+^ ions was synthesized by a conventional melt quenching method. The Fourier transform infrared (FTIR) results showed that PbO and Bi_2_O_3_ participate with PbO_4_ tetragonal pyramids and strongly distort BiO_6_ octahedral units in the glass network, which subsequently act as modifiers in glass structure. These results also confirmed the existence of both four and six coordination of germanium oxide in glass matrix.

## 1. Introduction

The optical fibers using heavy metal oxide glasses have good environmental stability and medium optical loss in comparison with fluorides, which makes them suitable for photonic applications in short distances [1]. Low cut-off optical phonon energy, which has appeared in lead-germanate glasses, decreases the nonradiative relaxation rate of erbium excited states leading to excellent upconversion efficiency [2]. In addition, bismuth-germanate glasses are increasingly used for applications in non-linear optics, optical switching and second harmonic generation (SHG) [3] especially because of their high linear and nonlinear refractive index [4], high thermal expansion, low transition temperature, and excellent infrared transmission [2].

To design a glass for specific applications, a basic understanding of host material is necessary and, therefore, analysis of their structure is useful. Recent infrared (IR) and Raman studies on manganese doped (100 − *x*)GeO_2_-*x*Bi_2_O_3_ system showed that Bi^3+^ ions incorporate in glass structure with deformed BiO_6_ octahedral units [5]. The distorted BiO_6_ octahedral groups participate in glass network by formation of non-bridging oxygens to modify the glass network. According to recent extended X-ray absorption fine structure (EXAFS) and vibrational study on *x*PbO-(1 − *x*)GeO_2_ system [6], at low lead content, lead ions act as modifiers in the germanate network, however, in PbO compositions higher than 40%, lead ions increasingly play a network former role in glass structure. Infrared spectroscopies are powerful techniques from which to infer structural information about glass material and hence many research groups are motivated to study different vitreous networks by IR spectroscopies [7,8]. In the present work, GeO_2_-PbO and GeO_2_-Bi_2_O_3_ binary and GeO_2_-PbO-Bi_2_O_3_ ternary systems as heavy metal oxide glass hosts with Er^3+^/Yb^3+^ dopants were studied. Fourier transform infrared (FTIR) spectroscopy was used to probe into the structure of the prepared glass samples.

## 2. Results and Discussion

### FTIR

**Figure 1:**
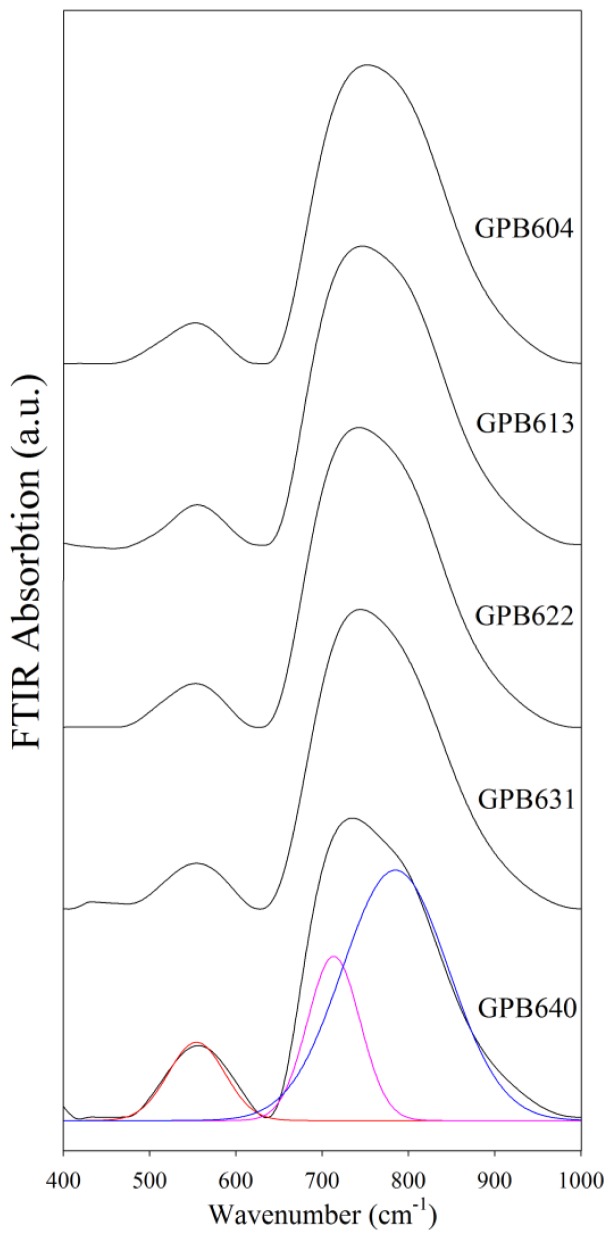
Fourier transform infrared (FTIR) spectrum of (GeO_2_)_0.6_(PbO)_0.4 −_ *_x_*(1/2Bi_2_O_3_)*_x_* glass of different compositions.

Figure 1 shows FTIR absorption spectra of GeO_2_-PbO-Bi_2_O_3_ glass where two envelops at around 500–600 and 700–800 cm^−1^ wavenumbers are present. Table 1 provides the wavenumbers of the deconvoluted bands. Absorption at 550 cm^−1^ indicates bending and symmetric stretching vibration of the Ge–O–Ge of GeO_4_ tetrahedral unit [9], Bi–O^(−)^ vibration bond of BiO_6_ unit [10] and antisymmetric bending vibration of Pb-O-Pb bond [11]. The band at 700–800 cm^−1^ wavenumbers was deconvoluted to peaks at 715 cm^−1^ and 785 cm^−1^. The first peak is related to stretching vibration mode of the Ge–O–Ge bond in GeO_6_ octahedral units of rutile germanium oxide [12]; the second is related to antisymmetric stretching vibration mode of the Ge–O–Ge bond in GeO_4_ tetrahedral units [9].

**Figure 2:**
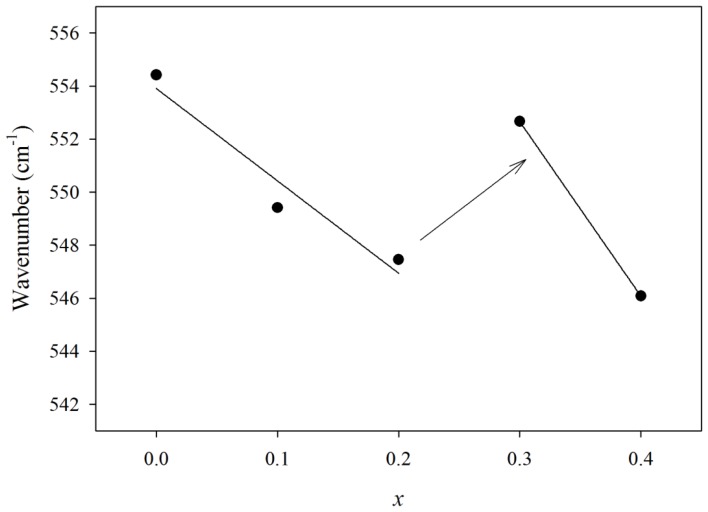
Dependence of FTIR wavenumber, located at about 550 cm^−1^, to bismuth composition of (GeO_2_)_0.6_(PbO)_0.4 −_ *_x_*(1/2Bi_2_O3)*_x_* glass. Bi-rich samples deviate to higher values.

FTIR absorption at 830 cm^−1^ is characteristic of the pyramidal BiO_3_ group [13]. Absence of this peak indicates that only BiO_6_ units participate in the glass structure. The peak at 470 cm^−1^ is related to symmetric bending vibration of Pb–O in PbO_4_ tetragonal pyramid (PbO covalent bond) and Bi–O bond bending of strongly distorted BiO_6_ octahedral units formed when both Pb and Bi act as network former in a glass matrix. Absence of this peak clearly indicates that the modification role of both lead and bismuth is predominant. Pb^2+^ ions in the germanate-lead oxide system act as a modifier in low PbO content with an increase of non-bridging oxygen, yet, participate in the glass network in high composition as PbO chains [6]. Based on Figure 2, the wavenumber at 550 cm^−1^ decreases gradually with increasing *x*. This is attributed to replacement of lead with bismuth ions where Bi^3+^ is surrounded by more oxygen than lead. Figure 2 shows two linear decreases of wavenumber in both Pb-rich and Bi-rich regions, but a deviation from the linear interpolation is observed when *x* changes from 0.2 to 0.3. This can be related to the appearance of Bi^2+^ and/or Bi^1+^ which have less oxygen in their surroundings in comparison with Bi^3+^ ions. Bi^3+^ is shared with three oxygens but Bi^2+^ and/or Bi^1+^ ions are surrounded with less oxygens. So Bi–O bonding should be stronger for Bi^2+^ or Bi^+^ ions in contrast to weaker bonding for the Bi^3+^ ion and appears in the deviation shown in Figure 2 to greater wavenumbers for GPB631 and GPB640.

The 700–800 cm^−1^ band was deconvoluted into two peaks at 715 and 785 cm^−1^ which are related to GeO_6_ and GeO_4_ units, respectively, and show the existence of both four and six coordination. Coordination number (*CN*), proposed by the Dachille and Roy study [14], relates the coordination number of cation “*T*” to the wavelength of the strongest infrared peak of the *T*–*X* stretching bond in TX_2_ compounds like SiO_2_, GeO_2_ and BeF_2_:

(1) $$K=\frac{CN\cdot \mu {\left(A_{T}+A_{X}\right)}^{1/3}}{Z_{T}Z_{X}\lambda^{2}}$$

where *A_T_* and *A_X_* are the atomic number of atoms *T* and *X*, respectively; *Z_T_* and *Z_X_* are valance of atoms *T* and *X*, respectively; *μ* is reduced mass and *K* is a constant. With *μ* in atomic mass units and *λ* in μm, Dachille and Roy [14] proposed the average value of *K* = 0.168. In this study, crystalline GeO_2_ precursor was used to determine the *K* value, supposing that all germanium atoms are 4-fold coordinated. The FTIR peak in 853 cm^−1^, substituted in the Dachille and Roy relation, yielded *K* = 0.163 which is in accordance with previous results [10,11].

**Figure 3:**
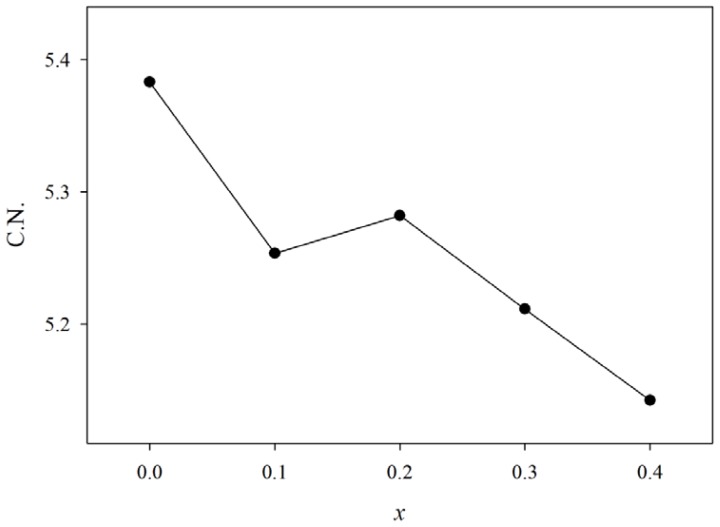
Dependence of coordination number of germanium to bismuth composition of (GeO_2_)_0.6_(PbO)_0.4 −_ *_x_*(1/2Bi_2_O_3_)*_x_* glass.

Figure 3 shows the average coordination number of Ge atoms in 6-fold coordination that was evaluated with Equation 1. The average coordination number decreases from 5.38 to 5.14 by increasing bismuth content, with a step-up between GPB631 and GPB622 showing the existence of *CN* values of both six and four, and a gradual change in germanium coordination from six to four with the sudden coordination change in the intermediate region.

## 3. Experimental Section

A series of GeO_2_-PbO-Bi_2_O_3_ glass samples were prepared by conventional melt-quenching method. High purity (more than 99%) GeO_2_, PbO, Bi_2_O_3_, Er_2_O_3_ and Yb_2_O_3_ precursors were used to synthesize [GeO_2_]_60_-[ PbO]_(40 −_ *_x_*_)_-[½Bi_2_O_3_]*_x_* with *x* = 0, 10, 20, 30, and 40 mol% and 0.5 and 1.5 wt% of Er_2_O_3_ and Yb_2_O_3,_ respectively. After mixing and grinding the precursors with the above concentrations and drying the mixture at about 300 °C, the mixture was heated at 1100 °C for 1 h. Then, the melt was quenched into a preheated cylindrical metal mold to obtain a transparent glass sample, and annealed at 420 °C. The samples were cut and polished for measurements.

FTIR spectroscopy was used to study the vibrational properties and structure of glass. The results were extracted from the Perkin-Elmer Spectrum-100 spectrometer, with a UATR accessory. Spectra were taken from 400 cm^−1^ to 1000 cm^−1^ and deconvoluted into Gaussian component bands.

## 4. Conclusions

The structure of glass host was studied by peak-deconvolution of FTIR spectra, which showed the existence of ionic Pb–O bonds in PbO_4_ tetragonal pyramids and Bi–O^(−)^ bonds in distorted BiO_6_ octahedral groups implying that Bi and Pb behave as modifier in glass. Deconvoluted spectra showed the existence of both four and six germanium coordination (GeO_4_ and GeO_6_ units) in all of the samples with variation of coordination number from 5.38 to 5.14 by increasing of bismuth content.

## Figures and Tables

**Table 1:** Deconvoluted wavenumbers and coordination number (CN) extracted from the Fourier transform infrared (FTIR) spectrum of (GeO_2_)_0.6_(PbO)_0.4 −_ *_x_*(1/2Bi_2_O_3_)*_x_* glass.

*x*	Composition (mol%)			Composition (wt%)		Sample Code	f_1_ (cm^−1^)	f_2_ (cm^−1^)	f_3_ (cm^−1^)	Coordination number
	
	GeO_2_	PbO	Bi_2_O_3_	Er_2_O_3_	Yb_2_O_3_					
0	60	40	0	0.5	1.5	GPB640	554	714	785	5.38
0.1	60	30	10	0.5	1.5	GPB631	549	720	791	5.25
0.2	60	20	20	0.5	1.5	GPB622	547	714	785	5.28
0.3	60	10	30	0.5	1.5	GPB613	553	717	788	5.21
0.4	60	0	40	0.5	1.5	GPB604	546	719	790	5.14
